# Unconscious Emotion and Free-Energy: A Philosophical and Neuroscientific Exploration

**DOI:** 10.3389/fpsyg.2020.00984

**Published:** 2020-05-21

**Authors:** Michael T. Michael

**Affiliations:** Underwood International College, Yonsei University, Seoul, South Korea

**Keywords:** unconscious emotion, free-energy, psychoanalysis, repression, alexithymia, hysteria, construal

## Abstract

Unconscious emotions are of central importance to psychoanalysis. They do, however, raise conceptual problems. The most pertinent concerns the intuition, shared by Freud, that consciousness is essential to emotion, which makes the idea of unconscious emotion seem paradoxical. In this paper, I address this paradox from the perspective of the philosopher R. C. Roberts’ account of emotions as concern-based construals. I provide an interpretation of this account in the context of affective neuroscience and explore the form of Freudian repression that emotions may be subject to under such an interpretation. This exploration draws on evidence from research on alexithymia and utilises ideas from free-energy neuroscience. The free-energy framework, moreover, facilitates an account of repression that avoids the homunculus objection and coheres with recent work on hysteria.

## Introduction

Freud appears ambivalent about emotion. On the one hand, he thought that it is “of the essence of an emotion that we should be aware of it, i.e., that it should become known to consciousness” (Freud, 1915/1957, p. 177). On the other hand, he frequently invoked unconscious emotion, such as “unconscious love, hate, anger, etc” (Freud, 1915/1957, p. 177). This ambivalence is reflected in an underdeveloped understanding of unconscious emotion in psychoanalysis today (Akhtar, 2013). The paradox suggested by Freud’s apparently conflicting stances has not yet been fully resolved. As such, a primary aim of this paper is to address this puzzle and provide an account that makes sense of both the reality of unconscious emotion and the intuition that consciousness is essential to emotion.

The topic of unconscious emotion is no mere side-issue to psychoanalysis. It reaches into the foundations of the psychoanalytic enterprise. An important reason for this is the role that unconscious emotion plays in psychopathology. In a recently published paper (Michael, 2018b; building on Edwards et al., 2012), I argued that unconscious emotion may lie at the core of hysterical symptoms. The argument, in brief, is that the repression of a memory can lead to the repression of accompanying emotion. As a result, when the memory, and hence the accompanying emotion, is unconsciously triggered, the patient may experience the bodily feelings generated by the emotion, but these feelings lack any explanation due to the unconsciousness of the emotion. Unexplained bodily feelings constitute prediction error – or free-energy – according to the Bayesian brain framework (see section Free-Energy and the Process of Repression). Without the availability of the correct explanation for these feelings, the brain attempts to construct a plausible alternative explanation, which in the right circumstances would be a symptom “belief.” This, in turn, can lead to the generation of symptoms of hysteria. If this account is correct, then it demonstrates the important role that unconscious emotion plays in the emergence of the kind of phenomena that psychoanalysis was first designed to address.

Given this, a broader aim of this paper is to address the Freudian paradox about unconscious emotion in a way that also sheds additional light on psychopathology. To do so I will invoke a philosophical account of emotion. This relates to a secondary aim of the paper, which is to bring a philosophical perspective into dialogue with psychoanalytic and neuroscientific perspectives. I believe that doing so, though challenging, is necessary to providing a more rounded and comprehensive understanding of the present issues. Emotion, though addressed by psychoanalysis and neuroscience, is not a concept that derives from these disciplines, but rather from our everyday psychological discourse, and philosophers have spent the last few decades analysing just such concepts. As such, the philosophy of emotion offers a prism through which a subtler understand of unconscious emotion may be attained.

The focus of the paper, to be more specific, will be on the *repression of the consciousness of emotion*. I use this cumbersome phrase to hone in on the form of repression at stake. Freud, despite his aforementioned comments, did speak about the repression of emotions, except that what he actually spoke about in this context was (chiefly) the *suppression of affect*. He wrote, “to suppress the development of affect is the true aim of repression and… its work is incomplete if this aim is not achieved” (Freud, 1915/1957, p. 178). There is a distinction, for Freud, between the suppression of affect and the repression of ideas, which is that “unconscious ideas continue to exist after repression as actual structures in the system *Ucs*., whereas all that corresponds in that system to unconscious affects is a potential beginning which is prevented from developing” (Freud, 1915/1957, p. 178). Thus, (full) suppression of affect cannot co-occur with (full) emotion, since on Freud’s account such suppression prevents the development of the emotion. What I wish to focus on instead, and which Freud, strictly, denied as a possibility, is the case where the emotion occurs – indeed, with felt consequences, as in the unexplained bodily feelings that, on my account, hysterics interpret as due to a symptom – but where *consciousness of* this emotion is prevented from arising due to repression.

In all probability, there are many gradations in the repression of emotion. Psychological defence against emotion, in other words, may bring about effects that fluctuate between numerous levels. These levels plausibly include the following: (1) suppression of the behavioural expression of an emotion that the agent is nevertheless acutely aware of; (2) repression of the consciousness of the emotion, as discussed above; and (3) full suppression of the emotion. As stated, our focus will be on the second level, since it is this which most relates to hysteria, and is the key to understanding Freud’s seemingly paradoxical comments on unconscious emotion.

## A Construal Account of Emotions

We begin with a philosophical account of emotion. The philosophy of emotion is important insofar as emotion is a concept that derives from commonsense psychological discourse, that is, the everyday discourse by which we make sense of our own and other people’s behaviour in terms of mental states such as belief, desire, and emotion. As such, it seems sensible to begin an endeavour to understand emotion by observing the constraints that our commonsense discourse sets on the concept. This is a motive for engaging in what analytic philosophers call “conceptual analysis,” the attempt to analyse concepts according to our most basic intuitions about their use in everyday language.

In order to motivate the account of emotion I will be presenting in this paper, I will first briefly offer some historical context. It is beyond the scope of the present paper to give anything other than a cursory review, but I will mention a couple of relevant developments within the recent history of the philosophy of emotions. One theory prominent in the late nineteenth and early twentieth century was the feeling theory, asserting that emotions are conscious feelings. A classic example of a feeling theory is the James-Lange theory (James, 1884; Lange, 1885), which posits that emotions are the perceptions of physiological changes in the body. Scientists and philosophers, however, soon observed that there were a number of problems faced by such theories. These include problems with accounting for the differences between emotions (since the feeling profiles of different emotions are often remarkably similar, while the feeling profiles of instances of the same emotion can differ widely), accounting for the rational dimension of emotions (drawing on the observation that emotions are subject to justification), accounting for the intentionality of emotions (in the sense of their being *about* some object), and accounting for the strong association between emotions and evaluations (e.g., fear seems to correspond in some way to evaluating an object as dangerous) (Scarantino and de Souza, 2018). Such problems do not entail that feelings theories should be dismissed, but they do require that such theories should be sophisticated enough to address these issues.

Motivated by the desire to deal with the problems brought against feeling theories, many philosophers moved in a different direction, developing judgement theories of emotion. Such theories assert that emotions are judgements (e.g., Neu, 1977; Solomon, 1980; Nussbaum, 1990). Fear, for instance, is the judgement that some object poses a danger to oneself. While such theories were popular for a while, they too encounter problems. An important issue is accounting for irrational emotions (Stocker and Hegeman, 1992). For example, an arachnophobe may judge a spider to be of no threat to him whatsoever, yet still fear it, suggesting there is a gap between judgement and emotion.

This brings us to the theory that I will be discussing in this paper: construal theory. The emergence of construal theories is a more recent development in the philosophy of emotions, arising as a result of criticisms of judgement theories. They offer an advance on judgement theories in that they can account for irrational emotions while still providing convincing solutions to the problems faced by feeling theories. In this paper, I will focus on a particular construal account that has been influential in the philosophical literature on emotion and provides a relatively simple yet plausible account of emotions. There are other versions of construal theory, but they have many of the same features as this one (see Lacewing, 2004, for a review). The account, proposed by Roberts (1988, 2003), is that emotions are concern-based construals.

In order to understand this account, we need to understand what Roberts intends by “construal.” The concept was inspired by a passage from the *Philosophical Investigations* (Wittgenstein, 1953, pp. 193–194), in which Wittgenstein talks about a sense of “sees” that is different from that of bare perception. He illustrates this sense with the famous duck-rabbit illusion: one can *see* the figure *as* a duck or *as* a rabbit. The seeing of some object, X, as something else, Y, is what Roberts calls a construal.

It is important to note that, though the concept of construal was inspired and is best illustrated by perceptual examples, it is not limited to such. As Roberts (1988, pp. 190–191) states, the elements of a construal, the X and Y terms, can be various – for example, they can be percepts, thoughts, images, concepts, or combinations of these. Elsewhere (Michael, 2018a, 2019b), I have argued that construal need not be conceptual in character – that is, the Y element need not involve concepts. Thus, for example, an infant may see a stranger as threatening even though she does not have the concept of threat. She sees the stranger through a set of experiences, involving perhaps various feelings, memories, imaginings, and so on, such that those experiences colour her experience of the stranger in a certain way, where this way is appropriately described as the aspect of being threatening.

Following Wittgenstein, Roberts takes a “family resemblance” approach to concepts, thus he does not hold that “construal” (or “emotion” for that matter) can be captured by a set of necessary or sufficient conditions. I agree, but nevertheless it will be helpful to adopt at least a working definition. To this end, and taking into account the point made above that construal need not be conceptual, I define construal as a way in which an intentional agent experiences or responds to some object, X, where this way of experiencing or responding can be appropriately described by phrases of the form “as Y” or “in terms of Y.”

According to Roberts, emotions are construals. A good way of understanding this is via Richard Lazarus’s influential appraisal theory of emotion. For Lazarus (1991), emotions involve appraisals, in the form of evaluations that he calls “core relational themes.” For example, anger involves appraising some object as having caused “a demeaning offence against me or mine”; fright involves “facing an immediate, concrete, and overwhelming physical danger”; and disgust involves “taking in or being too close to an indigestible object or idea” (Lazarus, 1991, p. 122). Lazarus holds that we make such appraisals in the form of judgements. On Roberts’ account, however, emotions need not involve judgements. Instead they are construals in the form of “see X as Y” where Y corresponds to a core relational theme. Thus, to be angry with a person is to see that person in terms of “a demeaning offence against me or mine” – that is, according to my definition, to experience or respond to the person in a particular way, where that way is appropriately described in terms of the given evaluation. To offer another example, the infant’s fear of the stranger is constituted (in part) by her seeing the stranger as threatening, that is, by her relating to him via a set of experiences or responses that can be collectively described as the aspect of being threatening^1^.

The other part of what constitutes an emotion, on Roberts’ account, is concern. By this he means a range of states which we can broadly term “desires” (relating to approach behaviours) or “aversions” (relating to avoidance behaviours)^2^. Emotions are concern-based construals, that is, construals filtered through desires or aversions. Such concerns enter into the construal as part of the Y-term: when I am angry with someone, I am not just seeing them in terms of having culpably offended against me, but also in terms of my concern not to be so offended against. Similarly, the infant is relating to the stranger via her aversion to threatening objects. Thus, an emotion is not simply an evaluative construal, but is rather one in which a concern is interwoven with the evaluation.

Among the merits of this account of emotion is that it makes sense of the explanatory role that emotions play in commonsense psychological discourse. Emotions typically explain subsequent behaviours and are explained by preceding events. We can explain my aggressive behaviour towards the person who has angered me, for example, by my concerned construal of the person as having offended against me, and we can explain this state in turn in terms of that person behaving towards me in a way that can plausibly be construed as offensive. The advantage that Roberts’ account has over judgement theories of emotion is that there are many cases in which one may have an emotion despite also having a judgement contrary to the evaluation associated with that emotion. These are, as we have seen, the “irrational emotions,” such as phobias. The arachnophobe may well judge that the spider before him is harmless, yet nevertheless be afraid of it. On Robert’s account, he is construing the spider as threatening, while judging it not to be. Elsewhere, I have elaborated on this distinction between judgement and construal, and illustrated how it can help solve numerous philosophical problems (Michael, 2018a).

In order to understand the power of this construal theory, it would be instructive to compare it with a recent feeling theory of emotion. The philosopher Prinz (2004) has offered a compelling update to the James-Lange theory of emotions, one that is sophisticated enough to address the problems brought against simple feeling theories. On this account, as in the James-Lange theory, the perception of bodily changes is constitutive of emotion. But, Prinz argues, this does not mean that we should give up on the idea that emotions are essentially evaluative. Rather, the perception of bodily changes can itself represent a core relational theme, so that emotions can be evaluative without being conceptual^3^. In other words, emotions are embodied appraisals: “They represent core relational themes, but they do so by perceiving bodily changes” (p. 68).

Prinz does not regard his theory as a construal theory, as he thinks that such theories assume the “conceptualisation” and “disembodiment” hypotheses. The conceptualisation hypothesis is the claim that “emotions require concepts” (p. 23). The disembodiment hypothesis is the claim that the components of emotion are “not identical to bodily changes or internal states that register bodily changes” (ibid.). I believe, however, he is wrong about the assumptions of construal theories. As we have seen, the version of construal theory I have presented does not assume the conceptualisation hypothesis, since the elements of a construal need not involve concepts. Also, it does not imply the disembodiment hypothesis: it allows that one can construe X as Y in virtue of a perception of bodily changes. Hence Prinz’s theory can be seen as a construal theory. Consider the example of one who feels fear upon seeing a snake. According to Prinz’s theory, the perception of the bodily changes brought on by the sight of the snake represents the core relational theme of danger, and it is this perception which constitutes the emotion of fear. On my interpretation of construal, this is the same as claiming that one construes the snake as dangerous, where this construal is in virtue of a perception of bodily changes^4^.

I offer this argument not as a means of endorsing Prinz’s theory, but to illustrate how a construal theory, broadly conceived, can accommodate sophisticated feeling theories, that is, ones that take into account the evaluative aspect of emotions. Construal, as I have defined it, is broad enough to encompass embodied non-conceptual construals. As such, it would be a mistake to describe construal theory, as some do (e.g., Smith and Lane, 2015), as a cognitivist theory. Though construals can be cognitive, they need not be. While resembling cognitions in respect of representing evaluations, they can consist solely of conscious feelings. This ideally suits them to an account of emotion, since, as we have seen, emotion has been analysed by some as a cognition and by others as a feeling, thus defining emotion in terms of construal allows for a compromise between these two positions.

Where Roberts’ account becomes most useful for our purpose of understanding unconscious emotion is in relation to the question of how we feel emotions. The word “feeling” can mean different things, but an important sense of the word is, according to Roberts (1988), captured by construal. This sense is different from that of the bodily feelings that may constitute the emotion. It is, rather, what Roberts calls a feeling of construed condition, that is, of taking oneself “to be in a certain condition” or “to have a certain property” (p. 185). For example, to feel excluded is to take oneself as being excluded (p. 186). It is this sense of feeling that, according to Roberts, is most relevant to the locution “feeling an emotion.” Thus, feeling our emotion involves a construal of our construal. Here is how Roberts (2003, p. 320) explains this idea:

Let us use subscripts to distinguish the two construals, a subscript 1 for the emotion and a subscript 2 for the feeling, and place brackets around the word “construal” to indicate that the ordinary subject does not experience his emotion in terms of the concept of a construal. Thus, to feel angry at Sally is to construe_2_ oneself as [construing_1_] Sally as having culpably offended in some matter that one strongly cares about. To feel proud of Nathan is to construe_2_ myself as [construing_1_] myself as increased in status because of Nathan’s attributes. To feel contrite is to construe_2_ myself as [construing_1_] myself as being or having done something contrary to some moral or quasi-moral standard that I am strongly concerned to meet.

So the feeling of an emotion is a (conscious) second-order construal, where what is construed is *oneself* in terms of a first-order construal. That is, when one feels an emotion one sees *oneself* in terms of *the way one is experiencing or responding to* some *object*^5^. This is a relationship between three elements: oneself, an object, and the way one is experiencing or responding to that object.

I will adopt Roberts’ account of feeling an emotion, though I prefer to call it the *consciousness of an emotion*. By adopting this terminology, I am not thereby implying that, in the absence of a second-order construal, an emotion cannot, in some sense, be conscious. Supposing, as we entertained earlier, that the emotion is constituted by the experience of bodily changes. Then the emotion is conscious even in the absence of a second-order construal insofar as those experiences are conscious. However, the person who has the emotion need not be conscious of it *as* her emotion, that is, as her taking some object in terms of a particular evaluation. She need not, in other words, be *reflectively* conscious of her emotion. It is this subtle distinction between different senses of consciousness that will help us address the Freudian paradox.

## A Neuroscientific Interpretation

Let me pause here to explore how these ideas might translate into terms more familiar to neuroscientists. In doing so, I caution against seeing the forthcoming discussion as an attempt to *reduce* Roberts’ account to neuroscientific terms. Roberts’ is a philosophical account, that is, an attempt to analyse the concept of emotion as it occurs in its “natural habitat” of commonsense psychological discourse. There is no good reason to think that either the concept of emotion or the concepts used to analyse emotion, such as construal, should correspond neatly to neuroscientific concepts. To borrow another idea from Wittgenstein, commonsense psychology and neuroscience may be different “language games” that cannot be fully reconciled. Nevertheless, I do not go as far as some philosophers in considering the two domains to be entirely autonomous. I believe, rather, that it is reasonable to expect a rough correspondence between what happens in the brain and what happens “in the mind,” so to speak, hence we can aspire to an approximate relation of ideas, as I hope the forthcoming discussion will illustrate.

Our focus will be on typical emotional episodes related to the basic emotions identified by Jaak Panksepp. Panksepp (1998) uses the term “emotional command system” to designate brain systems that, upon certain input, “generate instinctual behaviour output patterns” (p. 28) that can be associated with common emotions (or related states). He specifically identifies seven such systems, of FEAR, RAGE, LUST, CARE, PANIC/GRIEF, SEEKING, and PLAY. These systems form the basic, innately programmed response to relevant stimuli, though what makes a stimulus relevant and the precise nature of the response require individual learning. As Solms (2019, p. 8) puts it, “fear behaviours (freezing and fleeing), for example, are innate predictions; but each individual has to learn what to fear and what else might be done in response.” My purpose in drawing a connection with these ideas is to show how Roberts’ account of emotions may relate to neuroscientific accounts, though the sketch I present is a simplified one.

The starting point of a typical emotional episode, on this account, is a “stimulus” that “triggers” an emotional reaction^6^. This stimulus may be (the perception of) an external event or may be an internal event, such as a thought or memory. It may or may not involve a cognitive interpretation of the event (i.e., a judgement or cognitive construal). The proposal is that this stimulus triggers a basic emotional command system, thereby setting in motion physiological changes preparing the body for a particular kind of behavioural response – possibly alongside cognitive changes, such as changes in the “style and level of efficiency of cognitive process” (Damasio, 1994, p. 163) – pertinent to the basic emotion triggered. Such physiological changes may include changes in heart rate, blood pressure, breathing, metabolism, release of hormones, and so on. These physiological changes are experienced by the agent through conscious affective feelings. The feelings are, in the main, ones of valenced (i.e., pleasant or unpleasant) arousal (Barrett, 2017, p. 72), though the combination of effects generated by the stimulus may have numerous distinctive features^7^.

At this point, I will present one attractive possibility for interpreting Roberts’ account of emotion, though later I will challenge this interpretation. Continuing on from the above description, we note that the agent, in perceiving or thinking about the stimulus, will do so via the affective feelings generated by it, so that the stimulus is experienced in a particular way. It is therefore tempting to see these feelings (plus related memories, fantasies, beliefs, and so on) as thereby constituting the “colouring” with which some object (e.g., a person) is experienced. Because this “colouring” is valenced and related to specific behavioural tendencies, it serves as a particular perspective on or evaluation of the object. For example, the unpleasant arousal aiming at a flight or freeze reaction generated by the FEAR system in response to the perception of a snake constitutes (in part) the evaluative aspect of seeing something as threatening. In other words, the feelings accompanying inner bodily changes, through which the object that triggered this reaction is now being experienced, represent an evaluation of that object. Experiencing an object in terms of such feelings is, as we have already noted, an *embodied construal*: as a consequence (largely) of bodily changes, one is experiencing the object, X, in a certain way, where that way can be appropriately described using an “as Y” phrase with Y being an evaluation of X. Thus, we may conclude that the construal that constitutes an emotion (on Roberts’ account) is (usually) just such an embodied construal.

This interpretation has some nice features. First, it shows how a construal account captures the essential characteristics of emotion. The embodied construal described above is part of a causal chain that explains subsequent behaviour, and, when supplemented with an understanding of the predisposing tendencies of different kinds of stimuli, can be explained by preceding events. An embodied construal of this kind has the intentional (in the sense of being about some object) and evaluative character of emotion, in that it represents the evaluation of an object. It also has its motivational character, in that, in being a concern-based construal, it can predispose the agent towards certain actions (inherent in the behavioural preparedness triggered by the stimulus). Second, as we have already seen, the above interpretation also serves as a compromise between competing prominent theories of emotion, such as the James-Lange theory and appraisal theories, by utilising the idea that the experience of bodily changes is part of what constitutes an embodied appraisal. Third, in being a manifestation of a construal account, such an account is not tied to the basic or typical cases of emotional episodes described above, but potentially has wider applicability. Fourth, and most important for our purposes, this interpretation of emotion also captures a sense in which Freud’s assertion that emotions are always conscious might be true, for the affective feelings that are essential to how the object of the emotion is construed are conscious experiences.

However, though I am tempted by such an interpretation, I think it is not quite right. This is not to say it is completely false: experiencing an object via a set of conscious affective feelings is a construal, and it is part of the construal that constitutes an emotion. But it is not, or need not be, the whole of it. The construal which constitutes the emotion refers, rather, more wholly to *the organism’s response* to an object, beginning with the triggering of the emotional command system, up to and including the experiencing of the object via the conscious affects and other effects of this triggering. This entire organismic response is, I believe, what constitutes the construal that defines emotion, since the response as a whole (and not just some part of it) can be taken as the construed aspect^8^. Take for example the case of the perception of a snake triggering a fear response in an organism. The organism’s entire response to the snake, from the initial triggering of the emotional command system through to the experiencing of the snake via the arousal and other concomitant effects generated by the command system, constitutes that organism’s evaluative construal of the snake. Responding to an object in this way is as much an embodied construal as experiencing the snake in terms of the affective feelings generated: one is responding to an object, X (e.g., the snake), in a certain way, where that way can be appropriately described using an “as Y” phrase (“as threatening”), with Y being an evaluation of X^9^.

Henceforth I will refer to the first possibility, in which a construal and hence an emotion are constituted by the way an agent experiences an object, as a *narrow account* of construal and emotion, and the second possibility, in which a construal and hence an emotion are constituted by an organism’s response to an object, as a *broad account* of construal and emotion^10^.

Adopting a broader account of emotion renders the question of unconscious emotion less problematic. For on a narrower construal account, which focuses on how one experiences a certain object, consciousness is essential to the emotion. But on the broader account, one may be having an emotion without this necessarily having an effect on one’s conscious experience. In practice, this may make little difference, as an emotional episode will almost always influence how one experiences an object, but the conceptual distinction does at least allow for the possibility of entirely unconscious emotions. It seems to me, therefore, that the paradox of unconscious emotion, which Freud himself touched on, may arise as a result of adopting a narrower conception of emotion than is required. Nevertheless, as we proceed, it is worth having both accounts in mind, as the first account, even if incorrect, will help as understand why many, like Freud, have seen the idea of unconscious emotion as paradoxical.

## Levels of Emotional Awareness

It would be useful to connect the above ideas with an influential neuroscientific model of emotional consciousness, which I call the *Levels of Emotional Awareness* (*LEA*) model^11^. This model, inspired by Marr’s (1982) three-level theory of vision, has been most clearly articulated by Prinz (2004) and Lane et al. (2015). It posits that emotional consciousness is based on three levels of processing. The lowest level of the hierarchy pertains to local bodily states, that is, for example, changes in visceral states, changes in hormonal levels, and so on (Prinz, 2004, p. 213). Anatomically, Lane et al. (2015, p. 603) associate this level with the activity of brainstem nuclei. The intermediate level involves integrating these first-level processes into coherent patterns, ultimately “patterns of one’s entire bodily state across organs, muscles, and so forth” (p. 599). Anatomically, according to Lane et al. (2015), this level corresponds most closely with activity in the insula, “a predominantly sensory structure that registers and remaps bodily information and sensations into conscious somatic sensations” (p. 602)^12^. The highest level involves abstracting from particular patterns by categorising a range of such patterns under the same representation, that is, as “having the same emotional meaning” (ibid.). Lane et al. (2015) argue that this level of processing is associated with activity in the rostral anterior cingulate cortex (rACC), a region of the brain which specialises “in the representation of emotional meaning, particularly meaning that is concept-driven, by integrating highly processed interoceptive and exteroceptive information” (ibid.).

The LEA model may be useful in anchoring some of the ideas presented in the previous section. The first and second levels of processing described above, associated with activity in the brainstem and insula, correspond most closely with the affect and experience of bodily changes accompanying (and perhaps partly constituting) an emotion^13^. More importantly for our purposes, the highest level of processing in the LEA model, associated with the activity of the rACC, corresponds most closely with the second-order construal that constitutes the feeling of an emotion on Roberts’ account, for it is at this level that meaning is assigned to the emotional episode. As Lane et al. (2015) explain, “if the high-level of body state representation malfunctions then one will still experience and respond to bodily states, and other people will recognise them as expressions of emotion, but one will not experience them *as emotions*, be able to label them as such, or be able to use knowledge of their emotional meaning to plan to respond to them appropriately” (p. 599; authors’ emphasis).

Further support for the correspondence between second-order construal and the highest level of processing in the LEA model comes from Stevens (2016), who describes several lines of evidence suggesting that the consciousness of emotion is closely associated with rACC activity. For example, “studies examining the rACC region in alexithymia [a condition of reduced emotional awareness; see section Evidence From Alexithymia] show a pattern of hypoactivation” (p. 58). Also, in studies of different subtypes of depression, “a pattern emerges in which those that have awareness of their feelings show hyper rACC activation and those that are unaware of their feelings show hypo rACC activity” (p. 59).

Lane et al. (2015) also bring to attention another important dimension of the consciousness of an emotion, which is that it involves “situational appraisal.” They associate such appraisal with the ventromedial prefrontal cortex (vmPFC), stating that “one can think of this area as participating in the ongoing evaluation of emotional significance of stimuli in the environment in communication with cortical structures such as the insula and subcortical structures such as the amygdala, and generating representations of the emotional meaning of one’s situation” (p. 602). This kind of appraisal seems pertinent to second-order construal, since such is concerned with representing the meaning derived from one’s affective response to elements in the environment (i.e., one’s first-order construal of those elements). Focusing on such situational appraisal also brings to the fore the importance of context to the consciousness of emotion. The nature of an emotion cannot simply be read off the affective feelings it generates – indeed there may be no accurate mapping from quality of affective feeling to emotion (Barrett, 2017, p. 112). Rather an emotion needs to be understood in relation to a situational context, for, on the construal view of emotion, the emotion is an evaluation of some stimulus, where the nature of that evaluation depends on the wider circumstances in which that stimulus arose (Eickers et al., 2017). As we will see in section Free-Energy and the Process of Repression, this situational dimension can be important in determining why, in some cases, the emotion is repressed.

## Solution to the Freudian Paradox

As mentioned, the idea of unconscious emotion has been seen to present something of a paradox. This is because, in accord with Freud, many find it intuitive that consciousness is intrinsic to emotion. Yet this intuition has been challenged (e.g., Pulver, 1971) and the consensus within contemporary psychoanalysis is that emotion can be unconscious (Akhtar, 2013, pp. 14–15). Indeed, Freud himself acknowledged that talk of unconscious emotions is widespread in psychoanalysis:

But in psycho-analytic practice we are accustomed to speak of unconscious love, hate, anger, etc., and find it impossible to avoid even the strange conjunction, “unconscious consciousness of guilt,” or a paradoxical “unconscious anxiety” (Freud, 1915/1957, p. 177).

Freud does indeed make numerous references to unconscious emotion throughout his work (e.g., Freud, 1900/1957, p. 560, 1905/1957, pp. 56–57, 1909/1957, p. 240, 1910/1957, p. 144, 1911/1957, p. 63, 1919/1957, p. 231, 1933/1957, p. 139). So before we examine how repression works in relation to emotions, we need to first say more about this apparent paradox.

As I postulated in section A Neuroscientific Interpretation, one potential solution to the paradox is that Freud was adopting too narrow a view of emotion, one for which conscious experience is essential, whereas there is a broader view of emotion in which conscious experience is not essential. Hence emotion can be unconscious when taken in the broader sense, though is necessarily conscious when taken in the narrower sense.

But there is also another solution available, one that works even if we adopt only the narrow sense of emotion. This second solution to the paradox is suggested by Roberts’ account of what it is to feel an emotion. Recall that, for Roberts, having an emotion is having a first-order construal, while feeling an emotion is having a second-order construal, that is, a construal of oneself as construing some object in a certain way. This allows us to distinguish between two forms of consciousness: the conscious experiences that (partly) constitute the emotion and the *consciousness of* the emotion. In relation to the narrow interpretation of Roberts’ account, which focuses on embodied construal as a way of experiencing some object, this distinction can be stated as that between affective consciousness (feeling in the sense of affective feeling) and the consciousness of the emotion (feeling in the sense of feeling as a construed condition).

It is worthwhile saying a little more about the nature of the consciousness of an emotion. To do so we need to pay closer attention to the characteristics of the second-order construal (cf. Damasio, 1999). Whereas I have stated that a construal need not be conceptual, a second-order construal of the kind we are currently contemplating is conceptual. The experience that constitutes the emotion, itself an integration of various experiences of bodily change (and possibly of non-bodily changes), is construed as an instance of a particular kind of experience or response. Simultaneously, this is directly related to some object, so that it is a way of experiencing or responding to that object. At the same time this is understood as *one’s* way of experiencing or responding to the object. Such an integration seems only achievable by relating these elements conceptually. In the simplest case, one comes to construe these felt changes as, say, one’s anger at X, though such straightforward emotional labelling is not a requirement of the consciousness of an emotion^14^, but rather what matters is that one has a coherent and articulable perspective on the object.

## Repression of the Consciousness of Emotion

The above ideas readily lend themselves to the following characterisation of emotional repression^15^. The repression of the consciousness of an emotion is an active process that seeks to reduce attention on – or the precision of one’s model of (as we will see in section Free-Energy and the Process of Repression) – how one is experiencing or responding to the object of the emotion. This account of the repression of the consciousness of emotion has the advantage that it unproblematically allows that an agent can have an emotion, where that emotion is accompanied and partly constituted by conscious affective feelings, without being conscious of it, for the first-order construal that is the emotion need not be affected by the repression.

Such an account leads to some interesting reflections, which I will state in the form of a problem and suggested solutions. The problem is this: How, if an agent is experiencing the bodily changes involved in the emotion, can the repression of the second-order construal be sustained? For, it may be argued, the agent would surely need to interpret those experiences in some way.

There are at least two possible solutions to this problem. The first is that, though the correct second-order construal of the emotion is repressed, another, incorrect, construal can be constructed that offers an explanation of sorts for the given experiences. Indeed this possibility is, arguably, suggested by Freud:

In the first place, it may happen that an affective or emotional impulse is perceived but misconstrued. Owing to the repression of its proper representative it has been forced to become connected with another idea, and is now regarded by consciousness as the manifestation of that idea. If we restore the true connection, we call the original affective impulse an “unconscious” one. Yet its affect was never unconscious; all that happened was that its *idea* had undergone repression (Freud, 1915/1957, pp. 177–178).

There has been some discussion among psychoanalytic scholars as to how best to understand what Freud means by “proper representative” (e.g., Green, 2004; Herrera, 2010). The dominant view is that such a “representative” is a mental representation of the object of the emotion (Boag, 2012, p. 33), so that what Freud is talking about above is merely a displacement from one object to another. But there is another possible interpretation – which even if not exegetically correct, may be more theoretically appropriate – in line with my account. This is that the “proper representative” of the emotion is the second-order construal that constitutes the consciousness of an emotion. Thus, we can interpret Freud’s above assertion as that repression can cause an inaccurate second-order construal of one’s emotion to arise. Such a second-order construal can be inaccurate by misrepresenting the object of the emotion, as the standard interpretation asserts (corresponding to seeing one’s seeing X as Y as one’s seeing *A* as Y); or by misrepresenting the emotion as a different emotion by associating it with a different set of evaluative concepts (seeing one’s seeing X as Y as one’s seeing X as *B*); or even by misrepresenting the subject of the emotion as other than the self, thereby constituting projection (seeing one’s seeing X as Y as *S*’s seeing X as Y). Hence, in a more literal sense than that provided by the standard interpretation, an “emotional impulse is perceived but misconstrued.”

A second and more important answer to the question of how the unconsciousness of an emotion can be sustained in light of the conscious affective feelings it generates is that this is, in many cases, precisely the problem that leads to pathology. By repressing the second-order construal, one is left with unexplained experiences that constitute the prediction error that drives neurotic symptoms, as postulated by my Freudian version of the Bayesian account of hysteria (Michael, 2018b), described in the introduction. To say a little more about this, consider an agent who has repressed the consciousness of her emotion. Especially if she has increased bodily awareness (perhaps due to trait interoceptive sensibility, or increased body focus due to illness), she is likely to experience the bodily changes generated by the unconscious emotion while being unable to explain them. In which case, the repression becomes a “force” that compels her brain-mind towards alternative explanations. These alternative explanations may include a symptom “belief”^16^ (which can arise due to numerous factors, such as recent experiences with illness, cultural or other illness-related beliefs, or apt symbolic correspondences). As long as such a “belief” offers a plausible explanation, it may, due to the repressive need to keep attention away from the correct explanation, be favoured by Bayesian processes to the point where it becomes entrenched – that is, it is afforded a degree of precision the makes it immune to revision in the light of contrary sensory evidence. Such an entrenched symptom “belief” can thereby come to generate the symptom (see Michael, 2018b, for more details).

## Evidence From Alexithymia

The proposal that unconscious emotion involves the repression of a second-order construal of one’s emotion has support from work on alexithymia. Alexithymia is a condition characterised by an inability to gain awareness of one’s emotion and to express it in words^17^. It has often been cited in the philosophical literature on emotions as exemplifying unconscious emotion. For example, Lacewing (2007, p. 22) brings up alexithymia as “cases in which the subject reports no particular feelings at the time of the emotional episode,” stating that:

They generally disavow feeling emotions, and so they are also known as “alexithymics” (from the Greek for “having no words for emotion”). However, on the basis of how they interact with other people and the emotions they arouse in others, psychoanalysts argue that they do in fact have emotions, but that they are very out of touch with them.

The scientific literature on alexithymia suggests that, though alexithymics are not aware of their emotions (that is, according to Roberts’ account, they do not feel their emotions), they do feel the bodily sensations associated with the emotions. As Liemburg et al. (2012) put it, “alexithymia is characterised by difficulty to distinguish emotions from bodily sensations” (p. 660), so it is by failing to distinguish emotions from bodily sensations, rather than not feeling those sensations, that the problem (in part) arises. Moreover, the same authors found evidence for “a diminished connectivity within the DMN (default mode network) of alexithymic participants, in brain areas (such as the ACC) that may also be involved in emotional awareness and self-referential processing” (ibid.) – that is, just the kind of pattern we might expect in relation to a second-order construal that integrates the self with representations of one’s emotional state. These considerations cohere with the idea that the consciousness of an emotion is distinct from both having the emotion and from the consciousness of affect that may be partly constitutive of the emotion (at least, on a narrow account of emotion).

Interestingly, alexithymia has a high comorbidity with numerous psychiatric disorders:

Alexithymia has been associated with increased risk for psychosomatic complaints, anxiety disorders and depression. and the emotion regulation difficulties characteristic of alexithymia have been hypothesized to play a mediating role in these (ibid.).

Of particular relevance is the comorbidity with psychosomatic complaints, which, as characteristic of hysteria (or conversion disorder), may be a prime example of the pathology of repression (Michael, 2018b, 2019a)^18^. Gulpek et al. (2014) found that “[t]he level of alexithymia in conversion disorder patients, without any other psychiatric disorder, is higher than that of the healthy controls” (p. 300). In an independent study, Demartini et al. (2014) found that “alexithymia was present in 34.5% of patients with (functional motor symptoms)” (p. 1132)^19^. This suggests that the inability to be conscious of emotions can lead to pathological symptoms, indeed the very kind of symptoms that first led Freud on the path towards psychoanalysis. Accordingly, Demartini et al. go on to propose that “one hypothesis is that some patients misattribute autonomic symptoms of anxiety, for example, tremor, paraesthesiae, paralysis, to that of a physical illness” (p. 1132). This is very much in line with my own Freud-inspired proposal about the causes of hysterical symptoms (Michael, 2018b). It suggests that, just as the trait inability to be conscious of emotions can lead to hysterical symptoms in alexithymics, so too it might be that the repression-induced inability to be conscious of certain emotions can lead to hysterical symptoms in non-alexithymics^20^.

## Free-Energy and the Process of Repression

While the above account of the repression of the consciousness of emotion provides an outline of the form that such repression can take, we have yet to describe the process of repression itself. Coming up with such an account presents some *prima facie* problems, the most pertinent of which is avoiding a “homunculus” interpretation. This is the problem of explaining a particular mental process, such as repression, without treating some part of the brain as agent-like, in the sense of possessing psychological states and engaging in choices and actions – in other words, as an agent within the agent. Boag (2012) articulates this problem in his discussion of an influential account of repression based on Sullivan’s (1956) model of *selective inattention*, in which awareness involves intensive concentration on a target to the exclusion of other stimuli. Boag (2012) argues against such an account as follows (p. 195):

A single mind cannot be both exclusively aware of the target and also filtering incoming stimuli. Furthermore, the perceived “relevance” (or “irrelevance”) of stimuli is a judgement, which cannot preclude both awareness and evaluation of target material (though this need not be conscious itself). Consequently, selective inattention here requires that all incoming material be screened to determine whether it is or is not relevant.

This brings home the problem in providing a neuroscientific account of repression: what we require is an account of the process of repression that avoids treating it as the act of some inner agency, that is, some homunculus in the brain. It is here that the free-energy perspective can be of most assistance, as we shall see.

A second problem relates to the question of the purpose of repression. Why would the brain-mind repress the consciousness of an emotion? The consciousness of emotion is presumably an adaptive state, providing for a considerably more flexible response to one’s emotion than one would have if the emotion were unconscious. For example, it may be essential to the adaptive emotion regulation strategy of reappraisal (Subic-Wrana et al., 2014). Moreover, as we have discussed, it is probable that the absence of the consciousness of emotion often leads to psychopathology, such as hysterical symptoms. As such, it is, on the face of it, puzzling that there should be such an apparently maladaptive process as the repression of the consciousness of emotion. Once more, the free-energy perspective could be of assistance in addressing this question.

The free-energy perspective is useful for understanding what Freud called the “quantitative” dimension of mental activity. For Freud, that there is a quantitative dimension to mental activity is a fundamental tenet of his metapsychology, and he sought to understand all of the mind’s dynamics in terms of this factor (Freud, 1950 [1895]/1957). For example, he posits that “the use of the terms “unconscious affect” and “unconscious emotion” has reference to the vicissitudes undergone, in consequence of repression by the quantitative factor in the instinctual impulse” (Freud, 1915/1957, p. 178). Elsewhere he refers to this quantitative factor as “psychical energy” (e.g., Freud, 1900/1957, p. 568) or as the “sum of excitation” (e.g., Breuer and Freud, 1893-1895/1957, p. 86). Such quantitative expressions have fallen into relative disuse in psychoanalysis (Akhtar, 2013, p. 14), partly because of the difficulty in applying them, and partly because they have been subject to criticisms on the grounds of not having any obvious neurobiological underpinning (McCarley and Hobson, 1977). Recently, however, there has been a revival of interest in this aspect of psychodynamics due to the work of Karl Friston. According to Friston and his co-author Carhart-Harris, “the [Freudian] process of minimising ‘the sums of excitation’ is exactly the same as minimising the sum of squared prediction error or free-energy in Helmholtzian schemes” (Carhart-Harris and Friston, 2010, p. 1270). By this, they wish to equate the key idea of the Bayesian brain hypothesis, that the brain seeks to minimise prediction error (or, on Friston’s account, free-energy, which represents a bound on prediction error) with Freud’s fundamental “principle of constancy,” that the mind seeks to keep the level of psychical energy at a low and constant level.

The Bayesian brain hypothesis asserts that the brain is in the process of constructing hierarchically-organised multilevel “generative” models of the causes of sensory input, refining these in light of the input through Bayesian processes. At each level of the hierarchy of such a model, prediction units issue in predictions about the input from the level immediately beneath it, with the lowest level issuing predictions about the sensory input. These predictions are then compared, in prediction error units, to the input, and the difference, the prediction error, is fed up the hierarchy – thus the prediction error becomes the input to the next level. The inherent aim is to reduce the level of the prediction error, which can be done either by revising a model over a series of iterations (the basis of perception), or through bringing about movement that would change the sensory input in line with predictions (the basis of action). The theory is Bayesian because the processes by which predictions are generated correspond to those of Bayesian inference, in which the probability of a hypothesis is updated in light of evidence according to a formula involving the probability of the hypothesis prior to the given evidence – the “prior” – and the probability of the evidence given this hypothesis.

An important feature of this process is the role played by precision-weighting. This has to do with the degree of precision afforded to the prediction error versus the model at each level of the hierarchy. If more precision is given to the prediction error, then the model will be revised to a greater extent; if more precision is given to the model, then the prediction error will have less impact on revision. In cases where the model has an abnormally high precision, prediction error has little effect, and the representations given by the model become entrenched. This is, on my Bayesian account of hysteria (2018b), what purportedly happens with hysterical symptoms: a representation at a middle level of a hierarchical generative model, to the effect that the patient has a particular symptom, becomes entrenched due to excessively high precision being afforded to it, thereby coming to generate the symptom. On this account, the abnormally high precision is a consequence of the need to keep the real cause (an unconscious emotion) of changes in interoceptive input repressed.

The lowering of a model’s precision can also be highly consequential. An example of this is given by Prosser et al. (2018), in their free-energy model of psychopathy. In this model they postulate three levels of “belief,” corresponding to an unconscious self-schema (the lowest level), automatic conscious thoughts (the middle level), and high-level prior beliefs (the highest level). Importantly, the prior beliefs modulate the precision of the other two levels. It is through this modulatory connection that the authors account for psychopathic traits. For example, they model the psychopathic trait *lacks remorse* by having the prior beliefs lower the precision of a self-schema relating to feelings of shame or worthlessness. This leads to a relative decoupling of automatic conscious thoughts from such feelings, resulting in thoughts and behaviour that reflect the trait of lacking remorse. This nicely illustrates the pathological effects that the attenuation of precision can have on an agent.

I postulate that a roughly similar model can help explain the repression of the consciousness of emotions. In what follows I present only a preliminary sketch of such a model, as the details of a full model would be complex, taking us beyond the scope of the present paper. As in Prosser et al.’s model, there are, in this simplified model, three prominent levels at play. One, the lowest, corresponds to the experience of affect. The second, the middle level, corresponds to the second-order construal that constitutes the consciousness of the emotion. The third, the upper level, is a level superordinate to that of consciousness which modulates the precision of the levels beneath, that is, regulates consciousness. Such a superordinate level would correspond to a part of the Freudian ego, as it is the ego which, according to Freud, controls access to consciousness (Freud, 1926/1957, p. 95).

There is an important additional component to the model that has to do with the relation between the lowest level, pertaining to the experience of affect, and the upper level. In order to motivate this I turn to Connolly’s (2018) suggestion about how we can understand Freud’s “signal” theory of the triggering of repression from a free-energy perspective. Writing about situations of conflict between competing emotions, he proposes the following:

In essence, the updating of the generative model after the first experience of conflict means that the conflict state itself becomes reflected at a superordinate level of organisation through the altered precisions. The sensory stimuli which would previously have generated the conflict state of uncertainty now generates the defence state that privileges one response over another. An example of such a response might be an inhibitory response of the prefrontal cortex towards the limbic system, which now occurs without necessarily reexperiencing the initial conflict state, but is rather the result of a downward prediction encoded at a cortical level. In essence the conflict is now “predicted” and “resolved” through one stroke, through the precision weightings towards one pole of the conflict now avoiding the uncertainty of the conflict state (p. 12).

The important points remain applicable even in the absence of direct conflict between competing emotions. In place of conflict, we may substitute a traumatic experience – corresponding to large amounts of prediction error^21^ – brought on, in part, by the consciousness of an emotion. We may further suppose that, in the initial experience of the trauma, one of the means by which the prediction error was eventually reduced was by lowering the precision of the second-order construal that constitutes the consciousness of the emotion. If so, any future occurrences of that emotion could now come to generate the defensive response of lowering the precision on the second-order construal. That is, stimuli, such as a particular quality of affective feeling, that would previously have contributed to the generation of the second-order construal as an attempt to explain the feeling, now triggers (through prediction error feedback) a learned policy within the superordinate level of organisation (the third level of our model) for decreasing the precision of priors related to the consciousness of the emotion. This policy can be thought of as the operation of simultaneously predicting the re-experiencing of the trauma (hence large amounts of prediction error) and pre-empting it, in accord with the free-energy principle of minimising prediction error. Such a proposal, or an alternative that mirrors its general form even while differing in detail, enables us to avoid falling into the trap of positing homunculus-like agency to the brain, as there is no question of agency here, but rather simply a mathematically-governed process.

We can now turn to the second problem presented at the beginning of this section, recasting it in light of our free-energy model as follows: Why would the consciousness of an emotion elicit large amounts of prediction error? For, as mentioned, we may suppose that the consciousness of emotion plays an important role in the regulation of emotion, hence, if anything, would serve to reduce prediction error rather than increase it. An answer to the question is that the consciousness of an emotion can elicit high degrees of prediction error when it would be such as to lead to overwhelming negative affect, that is, affect that goes beyond that with which the brain can cope (hence warranting the epithet “traumatic”). Affect reflects prediction error (Solms and Friston, 2018), so overwhelming negative affect reflects a dangerous amount of prediction error.

This leads to an immediate follow-up question: Why would the consciousness of an emotion elicit such overwhelming negative affect? There are many possible answers, but I will focus on two that bear on important features of the consciousness of emotion.

The first possibility relates to my Bayesian account of hysteria. In this, the emotion whose unconsciousness leads to unexplained affect is unconscious due to being intimately connected with a repressed traumatic memory. We may relate this to the point made in section Levels of Emotional Awareness about the importance of situational context to the consciousness of emotion: for the emotion to become conscious, the situation that elicited that emotion would need to be accurately represented. In the cases we are considering, however, such situations have to do with memories that have been repressed, hence from the free-energy perspective have priors with low precision. Due to this repression they cannot be accurately represented, hence obstructing the construction of the second-order construal that would constitute the consciousness of the emotion. Indeed, going further, any attempt to make the emotion conscious would threaten the unconsciousness of the memory it is intimately associated with, so the policy of reducing the precision of priors associated with this traumatic memory may be extended to a policy of reducing the precision of priors associated with the consciousness of the emotion.

We may suppose that were this memory to become conscious, it would generate a degree of negative affect that would overwhelm the agent. Why so? On Freud’s theory, such memories are subject to repression on account not just of the emotion immediately generated by the memory, but also due to deeper negative emotions associated with it, ones that potentially reach down into highly aversive childhood experiences or infantile sexual fantasies. Thus, the consequences of such memories becoming conscious are an escalating series of negative effects, corresponding to escalating amounts of prediction error. In order to prevent such a consequence, a policy is formed that reduces the precision of any priors related to that memory and its accompanying emotion, thereby preventing any such mental phenomena from entering consciousness. Such reduction in priors might not be enough, however, to prevent all affective consequences: the initial emotion of the traumatic event could still be stimulated. But repression prevents the second-order construal that constitutes the consciousness of such emotion from being produced, thereby holding back or ameliorating the escalating series of negative effects that would re-traumatise the agent.

The second possibility for why the consciousness of an emotion would elicit overwhelming affect relates more directly to my account of the consciousness of emotion as a second-order construal. If this account is correct, then such consciousness involves seeing *oneself* in a certain way (as having a particular perspective on some object). It is, in other words, a *self*-construal. In so being, it makes the consciousness of an emotion liable to impact on one’s self-image, potentially bringing this into discord with one’s ego ideal^22^. The consequences of such could to be bring about excessively harsh superegoic judgements about the self, leading to potentially overwhelming negative emotions. It is in order to prevent such emotions that the higher-level policy to reduce lower-level precisions is triggered. This relates to Freud’s structural model of the mind, in which repression is seen to result from a conflict between superego and id. “Superego” here relates to high-level responses to one’s self-construal, and “id” relates to the initial instinctual generation of the emotion.

Thus, we may update our understanding of the process of repression as follows. Normally, the consciousness of an emotion is adaptive, as it helps in the regulation of the emotion (hence the reduction of prediction error). As such, normally the upper level of the generative model does not have a significant modulatory effect on the precision of the second level (or, perhaps, it increases the precision at that level). However, if in the past the agent has experienced overwhelming negative affect as a result (in part) of becoming conscious of the emotion in question, they develop, as a learned response, an alteration in the connections between the upper and the second level such that the precision of the second level is lowered in response to that emotion. In other words, a particular quality of negative affect has the effect of inducing the third level to lower the precision of the second level. The lowered precision at this level results in the failure of the emotion to attain consciousness. This leaves in its wake unexplained affect, but that is the price to pay for preventing the occurrence of the overwhelming affect which would have swamped the agent had the consciousness of emotion been allowed to develop.

## Discussion

The purpose of this paper has been to explore unconscious emotion in light of Freud’s seemingly paradoxical remarks, in which, on the one hand, he claimed that consciousness was essential to emotion, and on the other, he frequently invoked unconscious emotion. My answer to the apparent paradox is twofold, with both solutions emanating from a particular philosophical account of emotion, namely, Roberts’ account of emotions as concern-based construals. First, I pointed out an ambiguity in the concept of construal (reflecting an ambiguity in the concept of emotion) that allows us to give two slightly different accounts of emotion. In one, the narrow version, an emotion is constituted by the way one experiences an object, where this experience is coloured by the affect generated in response to the object. On this account, consciousness is essential to emotion. In the other, the broad version, an emotion is constituted by the organism’s response to an object, where this response can be described as an evaluation of that object. On this account, consciousness is not essential to emotion. This latter account thereby allows, at least conceptually, for the possibility of emotion devoid of conscious experience.

The second and more important solution to the paradox draws on Roberts’ account of what it means to feel an emotion. This account says that to feel an emotion is to experience oneself as construing an object in a particular way. If we equate this with the consciousness of an emotion, then we see how one can have an emotion without being conscious of it. This holds even if we adopt the narrow account of emotion described above, whereby consciousness – in the form of affective feelings – is essential to emotion.

This second solution opens up the possibility of the repression of emotion in a sense that goes beyond those which Freud spoke about, such as the suppression of the emotion. This is the repression of the second-order construal that constitutes the consciousness of the emotion. The existence of this form of repression is supported by evidence from alexithymia, a condition in which one can have an emotion without being conscious of it. It, moreover, complements my Freudian version of the Bayesian account of hysteria, for it is precisely due to the repression of the consciousness of an emotion that hysterics are left with the unexplained affect – hence prediction error – that leads to the formation of symptoms.

I further explored how this form of repression can be understood from a free-energy perspective, and thus addressed objections related to homunculi and the adaptiveness of repression. On this account, repression is the result of an affective signal that triggers a learned higher-order policy for reducing the precision of priors associated with the consciousness of the emotion that produced that affective signal. The policy has been learned as a result of past experiences, in which the consciousness of that emotion generated overwhelming affect, hence large amounts of prediction error. This generation of overwhelming affect may be explained in numerous ways, though I have focused on two explanations which draw on important facets of the second-order construal that constitutes the consciousness of an emotion. First, interpreting the way one is experiencing or responding to an object (i.e., the first-order construal) requires an understanding of the situational context. In the case where the emotion is interwoven with a traumatic memory, this would entail accessing this memory in way that could re-trigger the layers of affect underlying the trauma. Second, a construal of how one is construing things is a construal of one’s self, thus potentially bringing such a construal into discord with one’s ego ideal. This discord could generate overwhelming affect, hence large amounts of prediction error, due to superegoic responses to such conflict.

The exploration undertaken in this paper was an attempt to integrate philosophical, psychoanalytic, and neuroscientific viewpoints in addressing a number of interesting problems. The solutions I have offered to these problems are tentative, inspired more by an intention to show how different perspectives can inform each other than by an intention to provide definitive answers, so naturally there is much more to be said about all these issues. I hope to have shown, at least, that such an integration could be a fruitful source of ideas for making sense of the complexities of the psychodynamic aspects of mental functioning.

## Author Contributions

The author confirms being the sole contributor of this work and has approved it for publication.

## Conflict of Interest

The author declares that the research was conducted in the absence of any commercial or financial relationships that could be construed as a potential conflict of interest.

## Footnotes

1 On this account, where having an emotion is to construe X as Y, the X is what philosophers call the “particular object” of the emotion, and the Y is the “formal object” (Scarantino and de Souza, 2018).

2 This “concern” dimension of emotion resonates with Freud’s idea that an emotion is a manifestation of a drive (*Trieb*), since it accounts for the directedness of an emotion towards some need-based outcome.

3 This relies on Prinz’s theory of representation (Prinz, 2004, pp. 52–78), for which it suffices that a state is reliably caused by something (e.g., a core relational theme) and has been set up (by learning or evolution) to be reliably caused by that thing.

4 Here I am reproducing an argument I made in Michael (2019b).

5 The object of the emotion is whatever object the emotion is about. This is typically a particular (possibly non-existent) entity, though can also be a state of affairs.

6 The terms “stimulus” and “triggers” are perhaps misleading, as perceptions, thoughts, emotions, and so on, are, on a free-energy account, the result of simultaneous cascading predictions (Barrett, 2017). Nevertheless, the terms are expositorily convenient, and, since our focus is not on the initiation of emotion but its subsequent course, I will continue to use them in the forthcoming discussion.

7 Here, in order not to complicate matters, I skate over the distinction between what Prinz calls the “perception of bodily changes” and the affect, that is, the conscious feeling on which this perception is based. Solms and Friston (2018) argue that affect is the subjective manifestation of forebrain arousal by the brainstem, triggered by prediction error. I suggest that this may relate to Prinz’s “perception of bodily changes” through a construal: I feel X (some body region) in terms of Y (affect). If this is correct, then the “perception of bodily change” is itself a construal, but different from the construal that is the emotion (though this may construe *in virtue* of such perceptions).

8 This is in line with a free-energy account, since the action plan generated by the emotional command system may be seen simultaneously as an inference about the causes of sensory input. That is to say, as the perception of the “stimulus” is being constructed, the brain is at the same time predicting the body’s needs in response to this stimulus, hence in preparing a particular action plan (e.g., for a “flight” response) it is also thereby evaluating the stimulus (say, as threatening). The action plan and the evaluation are not two distinct states, but rather are two sides of the same coin.

9 The response meets Prinz’s (2004) criteria for a representation (see footnote 3), since it is reliably caused by a core relation theme (such as danger) and has been set up by evolution and learning to be so reliably caused.

10 The narrow account of construal fits best with the phrase “sees X as Y,” while the broad account of construal fits best with the phrase “takes X as Y.”

11 The name derives from Lane et al.’s (1990) “Levels of Emotional Awareness Scale.”

12 It should be noted that the role of the insula in emotional awareness is contested (Damasio et al., 2013). While it is widely believed that the insula normally plays a role in emotional awareness, it is not yet known precisely what this role is, and it may be that other brain areas can perform similar functions in cases where the insula has been damaged (ibid., p. 844).

13 Solms (2013, 2019) and Solms and Friston (2018) make a powerful case that affect is generated by the brainstem, while perceptions relate to cortical regions. This seems consistent with Prinz’s (2004) account of the perception of bodily changes, which he associates with the insula (p. 215). Thus, on this view, the first level of the LEA corresponds to affect, and the second level to the perception of bodily changes in terms of such affect (see footnote 7).

14 Roberts (2003, p. 321) writes, “I do not suggest that we cannot feel an emotion (in my sense of “feel”) unless we have a name for the emotion. The important thing is that we have ways of conceptualising ourselves, and I should think it obvious that we have a lot more concepts than we have concept words.”

15 In using the term “repression” I am following Freud’s usage in *The Unconscious* (Freud, 1915/1957, p. 178).

16 “Belief” here is not to be understood in the usual sense, as a propositional attitude. It is rather a representation encoded by the activity of a population of neurons, occurring as part of a hierarchical model of the causes of sensory input (see section Free-Energy and the Process of Repression).

17 See Taylor and Bagby (2013) for a more in-depth understanding of the alexithymia construct, including its historical background and empirical grounding. For a psychoanalytic perspective on the condition, see McDougall (1982, 1989).

18 The relationship between alexithymia and psychosomatic disorders has been recognised for some time, for example by Nemiah (1977) and McDougall (1982).

19 A more recent designation of hysterical symptoms is as “functional neurological symptoms,” of which a prevalent kind are “functional motor symptoms.”

20 It may be that there is a yet closer relationship between alexithymia and repression. Taylor et al. (2016) have noted a parallel between alexithymia and emotional repression, particularly with respect to Freud’s notion of primal repression. The association between alexithymia and repression is also in line other studies: the imaging work of Liemburg et al. (2012, p. 665) indicates that alexithymia is associated with “higher connectivity in right-sided prefrontal regions” of the brain – regions that may correspond with repressive processes (Depue et al., 2007; Kikuchi et al., 2010) – a finding supported by more recent work (Kim et al., 2020).

21 As Hopkins (2016) observes, “complexity [equal to free-energy plus accuracy, a measure of the predictive success of a model] is conceptually linked with *emotional conflict* and *trauma.*” He goes on to explain that “experiences are rightly regarded as traumatic when the emotional adjustments (complexity) required for integrating them into thought and action are greater than the brain can manage.”

22 McDougall (1982) describes the conditions under which an ego ideal that is pathological in relation to emotions may develop. For example, as one of her alexithymic patients expressed, “In our family it was forbidden to be sad, or angry, or in need of anything. I still get confused if I try to grasp what I am feeling” (p. 84).
